# Description of a new *Pselaphodes* Westwood (Coleoptera: Staphylinidae: Pselaphinae) from Vietnam

**DOI:** 10.3897/BDJ.7.e46327

**Published:** 2019-10-03

**Authors:** Rostislav Bekchiev, Zi-Wei Yin, Manh Quang Vu, My Ha Tra, Hanh Hoang Nguyen

**Affiliations:** 1 National Museum of Natural History, Bulgarian Academy of Sciences, 1 Tsar Osvoboditel Blvd., 1000, Sofia, Bulgaria National Museum of Natural History, Bulgarian Academy of Sciences 1 Tsar Osvoboditel Blvd., 1000, Sofia Bulgaria; 2 Lab of Environmental Entomology, College of Life Sciences, Shanghai Normal University, 100 Guilin Road, Shanghai 200234, Shanghai, China Lab of Environmental Entomology, College of Life Sciences, Shanghai Normal University 100 Guilin Road, Shanghai 200234, Shanghai China; 3 Hanoi National University of Education (HNUE), 136 Xuan Thuy Rd., DHSP Cau Giay, Hanoi, Vietnam Hanoi National University of Education (HNUE) 136 Xuan Thuy Rd., DHSP Cau Giay, Hanoi Vietnam; 4 Ho Chi Minh City University of Food Industry, 140 Le Trong Tan St., Tan Phu, Ho Chi Minh, Vietnam Ho Chi Minh City University of Food Industry 140 Le Trong Tan St., Tan Phu, Ho Chi Minh Vietnam; 5 Institute of Ecology and Works protection, Hanoi, Vietnam Institute of Ecology and Works protection Hanoi Vietnam

**Keywords:** Asia, Indochina, Tyrini, taxonomy, new species

## Abstract

**Background:**

A new species of the genus *Pselaphodes* Westwood, *P. fansipanensis* Bekchiev & Yin sp. n., is described from northern Vietnam. The unique modification of the antennomeres 9–10 of the male readily separates the new species from all known congeners.

**New information:**

New Pselaphodes species from Vietnam

## Introduction

The Pselaphinae (Coleoptera: Staphylinidae) fauna of Vietnam is poorly studied. Until now, 112 species (Bekchiev 2010, Nomura 2013, Nomura and Pham 2019) have been known from the country. The diversity of habitats and environmental conditions in Vietnam implies that the diversity of pselaphine fauna may be much greater. A total of 65 species of *Pselaphodes* (Tyrini) have been described, all from the Oriental region - China, Nepal, India, Sri Lanka, Thailand, the Philippines and East Malaysia (see Huang et al. 2018 for checklist and distributional map) and two additional unnamed species were recently reported from Vietnam (Nomura and Pham 2019).

In September 2018, a short expedition to southern and northern Vietnam was organised by the Institute of Ecology and Works Protection (Hanoi) and the National Museum of Natural History (Sofia), giving the opportunity to collect rich zoological material. One of the visited localities was Fansipan Mountain and, especially, the Fansipan Summit. It is the highest (3,143 metres a.s.l.) mountain in the Indochinese Peninsula (including Vietnam, Laos and Cambodia), hence its nickname "the Roof of Indochina”.

Included in the material was a new *Pselaphodes* species, which is described below.

## Materials and methods

**Figure 1.**
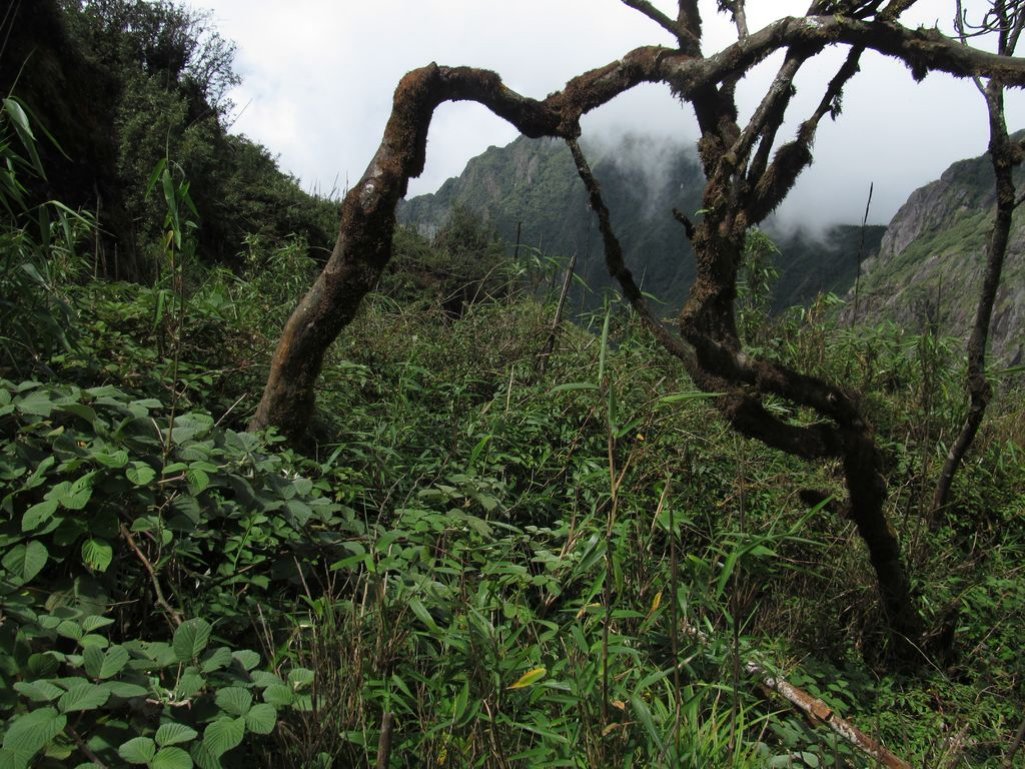
Fansipan Summit - habitat of *Pselaphodes fansipanensis* sp. n.

The material was collected in an open habitat with shrubs and grasses (Fig. 1) by soil and litter sifting.

Specimens were examined by Zeiss Stemi 2000C stereo-microscopes. Male genitalia and other dissected parts were studied using a Zeiss transmitted-light microscope at magnifications up to 500x. Genital segments were dissected and treated with potassium hydroxide (KOH). The dissected parts were mounted in Euparal and pinned with the relevant specimen.

The following acronyms are used in the text: BL—length of the body (= HL + PL + EL + AL); HL—length of the head from the anterior clypeal margin to the occipital constriction; HW—width of the head across eyes; PL— length of the pronotum along the midline; PW—maximum width of the pronotum; EL—length of the elytra along the suture; EW—maximum width of the elytra; AL—length of dorsally visible part of the abdomen along the midline; AW—maximum width of the abdomen; NMNHS – National Museum of Natural History, Sofia, Bulgaria

The type specimen is provided with a red printed label: ”HOLOTYPUS”, “*Pselaphodes fansipanensis* sp. n.”, “R. Bekchiev & Zi-Wei Yin, 2019 “ .

## Taxon treatments

### Pselaphodes fansipanensis

Bekchiev & Yin sp. n.

D09F6ED4-1593-5404-953B-D5FB98029570

urn:lsid:zoobank.org:act:EB4A1A24-6782-4926-97E6-08CCFF8B1B79

#### Materials

- **Type status:** Holotype. **Occurrence:** recordedBy: R. Bekchiev, N. Simov, I. Dedov, P. Beron; individualCount: 1; sex: male; lifeStage: adult; **Taxon:** scientificNameID: Pselaphodes fansipanensis; higherClassification: Coleoptera; Staphylinidae; Pselaphinae; class: Insecta; order: Coleoptera; family: Staphylinidae; genus: Pselaphodes; specificEpithet: fansipanensis; taxonRank: species; **Location:** locationID: Fansipan peak; higherGeographyID: Lao Cai Province; higherGeography: Vietnam; continent: Asia; verbatimElevation: 2992 m; verbatimLatitude: 22.30560; verbatimLongitude: 103.77625; decimalLatitude: 22.30560; decimalLongitude: 103.77625; **Identification:** identifiedBy: Rostislav Bekchiev, Zi-Wei Yin; dateIdentified: 2019; **Record Level:** institutionID: National Museum of Natural History-Sofia; institutionCode: NMNHS

#### Description

**Figure 2.**
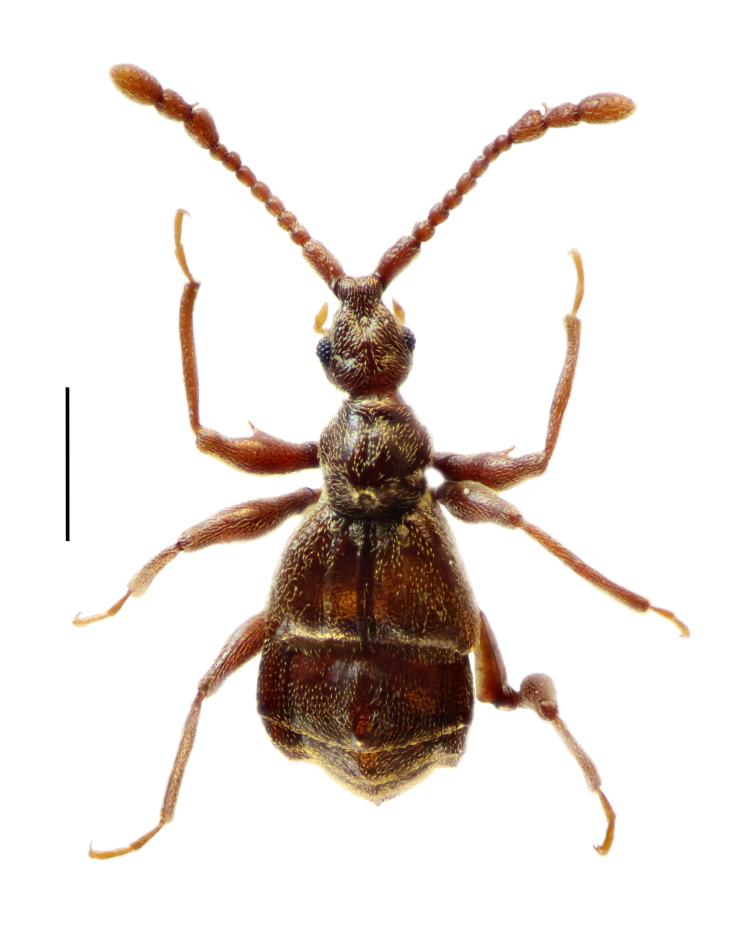
*Pselaphodes fansipanensis* sp. n. - habitus. Scale bar: 1.0 mm.

Male (Fig. 2). Body reddish-brown, covered with short golden setae, BL 3.14 mm. Head slightly longer than wide, HL 0.62 mm, HW 0.56 mm; each eye composed of about 40 facets.

**Figure 3.**
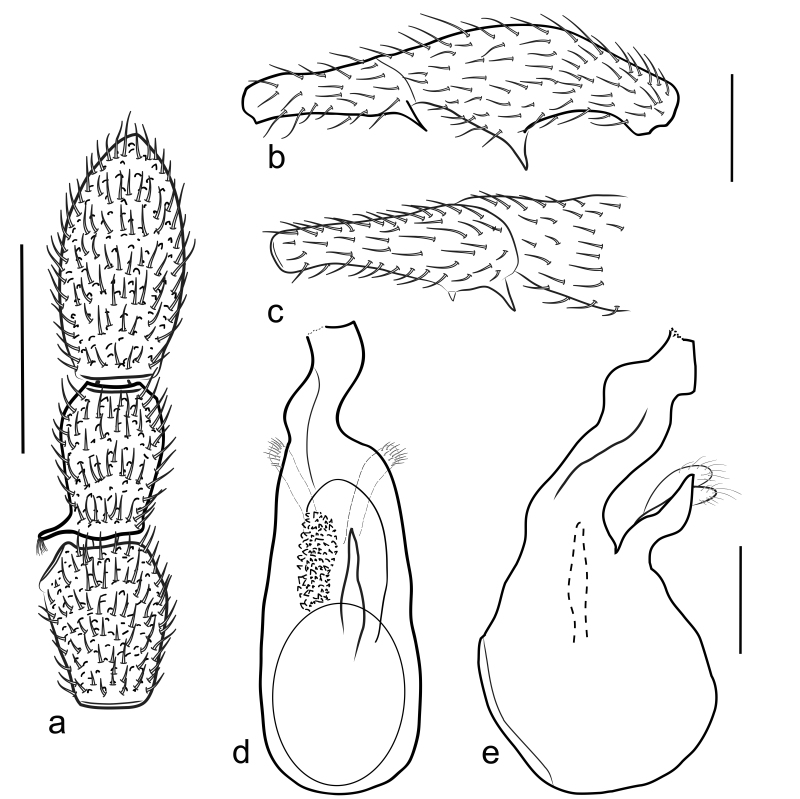
*Pselaphodes fansipanensis* sp. n. - a) antennal club; b) protrochanter and profemur; c) mesotrochanter; d, e) aedeagus - dorsal and lateral view. Scale bar: 0.2 mm.

Antennomeres (Fig. 3a) 9–11 forming distinct club, antennomere 9 modified, with disc-shaped process at apex, antennomere 10 with thin, elongate protuberance at base. Pronotum as long as wide, PL 0.62 mm, PW 0.63 mm, coarsely punctate and regularly pubescent, with lateral margins rounded at apical third and then narrowing apicad. Elytra wider than long, EL 0.78 mm, EW 1.18 mm. Metaventral processes short, triangular, curved ventrally at apices in lateral view. Protrochanter with one thin projection, profemur (Fig. 3b) with one large, bluntly triangular ventral projection; protibia simple; mesotrochanter with two ventral spines, one minute and one larger, one minute and one distinct ventral spines (Fig. 3c); mesofemur and mesotibia simple; hind legs simple. Abdomen broad at base and narrowed apically, with large hump at middle of tergite IV; AL 1.12 mm, AW 1.24 mm.

Length of aedeagus 0.77 mm, median lobe broad and asymmetrical, parameres elongate, endophallus with one elongate sclerite (Fig. 3d, e); the apical part of the median lobe was broken and lost during the preparation of the specimen.

Female. Unknown.

#### Etymology

The new species is named after Fansipan Summit, the type locality of the new species.

#### Distribution

North Vietnam: Lao Cai Province.

#### Taxon discussion

The new species can be readily separated from all other members of the genus by the unique modification of the antennomeres 9–10 and the short, triangular metaventral processes of the male, as well as the shape of the aedeagus.

## Supplementary Material

XML Treatment for Pselaphodes fansipanensis
